# Chinese Visceral Adiposity Index Is Associated With Incident Renal Damage in Patients With Hypertension and Abnormal Glucose Metabolism: A Longitudinal Study

**DOI:** 10.3389/fendo.2022.910329

**Published:** 2022-07-14

**Authors:** Mengyue Lin, Nanfang Li, Mulalibieke Heizhati, Lin Gan, Qing Zhu, Ling Yao, Mei Li, Wenbo Yang

**Affiliations:** Hypertension Center of People’s Hospital of Xinjiang Uygur Autonomous Region, National Health Committee Key Laboratory of Hypertension Clinical Research, Key Laboratory of Xinjiang Uygur Autonomous Region “Hypertension Research Laboratory, Xinjiang Clinical Medical Research Center for Hypertension (Cardio-Cerebrovascular) Diseases, Xinjiang Hypertension Institute., Urumqi, China

**Keywords:** diabetes, hypertension, Chinese visceral adiposity index, renal damage, cohort

## Abstract

**Objective:**

To evaluate the association between Chinese visceral adiposity index (CVAI) and incident renal damage and compared its predictive power with that of other visceral obesity indices in patients with hypertension and abnormal glucose metabolism (AGM).

**Methods:**

This retrospective cohort consecutively included patients with hypertension and AGM who did not have renal damage at baseline. Renal damage was defined using the estimated glomerular filtration rate (eGFR) and urine protein. Multivariable Cox regression analysis was used to evaluate the association between CVAI and incident renal damage. Restricted cubic splines were used to determine the shape of the association. The predictive power of the CVAI was examined and directly compared with other indices, including the VAI, body mass index (BMI), waist circumference (WC), and waist-to-height ratio (WHtR), using the area under the receiver operating characteristic curve (AUC) and C-index.

**Results:**

In total, 2,033 patients with hypertension and AGM were included. During a median follow-up of 2.6 years, the incidence of renal damage was 31.5, 48.9, 56.8, and 67.5/1,000 person-years across the quartiles of CVAI. Compared with the first quartile, the risk of renal damage was higher in the second (hazard ratio (HR) = 1.36 [95% CI: 0.93−1.97]), third (HR = 1.57 [95% CI: 1.09−2.27]), and fourth (HR = 1.65 [95% CI: 1.11−2.44]) quartiles (*p* for trend = 0.011). A linear dose–response association was observed. Sensitivity and subgroup analyses confirmed the robustness and consistency of the results. In terms of predictive power, the CVAI had the highest AUC and C-index values.

**Conclusions:**

CVAI is positively associated with renal damage risk in a linear dose–response pattern and has the best performance in predicting incident renal damage in patients with hypertension and AGM. The CVAI may serve as a reliable indicator for identifying patients at a high risk of renal damage.

## Introduction

Chronic kidney disease (CKD) has been recognized as a major public health issue due to its high prevalence and strong association with cardiovascular events and premature death (1). The prevalence and incidence of CKD are increasing as an ongoing epidemic of metabolic diseases, such as hypertension, abnormal glucose metabolism (AGM), and obesity (2). We recently found in a population-based study that the prevalence of kidney dysfunction in patients with hypertension and diabetes was higher than that in those with either hypertension or diabetes alone (3). Notably, the prevalence of AGM, including diabetes (12.4%) and prediabetes (38.1%), is more than 50% among Chinese adults (4). Given the synergistic effect of hypertension and hyperglycemia on renal damage (5), it would be beneficial for disease management to focus on patients with hypertension and AGM (6). However, traditional risk factors fail to fully explain the increased risk of renal damage in this patient population (7).

Studies have shown that visceral obesity is associated with organ injury, resulting in an increased risk of hypertension, carotid atherosclerosis, diabetes, and kidney disease (8–10). MRI and CT are the two most sensitive methods for measuring visceral fat. However, the use of both procedures for screening large populations is infeasible because of expensive equipment and ionizing radiation (11). Recently, Xia et al. established a Chinese visceral adiposity index (CVAI) to estimate visceral adiposity and predict metabolic disorders (12). CVAI has been shown to outperform other visceral obesity indices in predicting prediabetes, diabetes, and carotid plaque in the Chinese population (9, 13). In addition, several studies have reported an association between obesity and CKD, with visceral obesity appearing to be more closely related to kidney impairment (14–16). However, the association of CVAI with the risk of renal damage has not been reported, especially in patients with hypertension and AGM, a high-risk group for kidney disease.

Therefore, this study aimed to evaluate the association between CVAI and the risk of renal damage in patients with hypertension and AGM, based on a longitudinal cohort. We also compared the predictive power of the CVAI with other indices to determine whether the CVAI could be a better indicator for identifying high-risk individuals.

## Methods

### Study Population

The study population was recruited from the Hypertension Center of the People’s Hospital of Xinjiang Uygur Autonomous Region between January 2012 and May 2019. Inpatients aged ≥18 years with hypertension and AGM were consecutively included. Exclusion criteria were diagnosis of secondary hypertension (primary aldosteronism, adrenal tumor, Cushing syndrome, pheochromocytoma, and polycystic ovary syndrome), history of cardiovascular events within the last 3 months (including myocardial infarction, heart failure, stroke, unstable angina, coronary revascularization, and coronary bypass surgery), or malignant tumor. In addition, patients with CKD at baseline were also excluded. A total of 2,459 patients with hypertension and AGM and free of CKD at baseline were initially identified, and 2,033 of them completed follow-up at least once and were finally analyzed. This study was performed in accordance with the Declaration of Helsinki and approved by the Ethics Committee of the People’s Hospital of Xinjiang Uygur Autonomous Region.

### Data Collection

Baseline information was extracted from the medical electronic system, including age, sex, height, weight, waist circumference (WC), cigarette consumption (yes or no), alcohol intake (yes or no), blood pressure (BP), fasting plasma glucose (FPG), glycosylated hemoglobin (HbA1c), total cholesterol (TC), triglyceride (TG), high-density lipoprotein cholesterol (HDL-C), low-density lipoprotein cholesterol (LDL-C), blood urea nitrogen (BUN), uric acid (UA), serum creatinine (Scr), duration of hypertension, type of AGM (prediabetes or diabetes), plasma aldosterone concentration (PAC), plasma renin activity (PRA), and medication use (antihypertensive, lipid lowering, and hypoglycemic drugs). Body mass index (BMI) was calculated as weight (kg) divided by height (m) squared. The waist-to-height ratio (WHtR) was calculated as the WC divided by height.

Seated BP at the time of hospitalization was measured in the upper arm after patients rested quietly for at least 10 min with a mercury sphygmomanometer using international recommendations (17). The mean values of two measurements were recorded and used for the analysis. WC was measured at the midway level between the lower rib margin and the iliac crest in the midaxillary line, with the participants standing with their feet 25–30 cm apart.

### Definition of Diseases and Obesity Indices

Hypertension was defined as systolic BP (SBP) ≥140 mmHg and/or diastolic BP (DBP) ≥90 mmHg or the use of antihypertensive drugs. AGM includes prediabetes and diabetes. Prediabetes was defined as FPG ranging from 6.1 to <7.0 mmol/L or 2-h postprandial glucose ranging from 7.8 to <11.0 mmol/L. Diabetes was defined if there was a previously confirmed diagnosis, or FPG was ≥7.0 mmol/L, or 2-h postprandial glucose was ≥11.1 mmol/L. The estimated glomerular filtration rate (eGFR) was calculated using the simplified modification of diet in renal disease (MDRD) equation based on data from Chinese adults (18). Urine protein levels were determined using urine dipstick results (−, ±, 1+, 2+, and 3+). Renal damage was defined as an eGFR < 60 ml/min/1.73 m^2^ and/or the presence of proteinuria (≥1+). CVAI and visceral adiposity index (VAI) were calculated as follows (12, 19):


$$\text{CVAI}\left(\text{men}\right)=-267.93+0.68\times \text{age}+0.03\times \text{BMI}+4.00\times \text{WC}+22.00\times {\text{log}}_{10}\text{TG}-16.32\times \text{HDL}-\text{C}.$$

$$\text{CVAI}\left(\text{women}\right)=-187.32+1.71\times \text{age}+4.23\times \text{BMI}+1.12\times \text{WC}+39.76\times {\text{log}}_{10}\text{TG}-11.66\times \text{HDL}-\text{C}.$$

VAI(men)=(WC/39.68+[1.88×BMI])×(TG/1.03)×(1.31/HDL).

VAI(women)=(WC/36.58+[1.89×BMI])×(TG/0.81)×(1.52/HDL).

### Follow-Up and Outcome

The outcome of this study was new-onset renal damage during follow-up. Follow-up data were obtained using annual health checkups or hospital readmissions. An examination time ≥3 months after baseline was considered valid. Only the first outcome was used for the analysis if a participant experienced the outcomes more than once during the follow-up period.

### Statistical Analysis

Baseline characteristics were described according to CVAI quartiles. Continuous variables were presented as mean ± SD or median (interquartile range [IQR]) according to the normality test results and compared between groups using analysis of variance or non-parametric Kruskal–Wallis H test. Categorical variables were summarized as numbers and percentages and compared between groups using Pearson’s chi-square test.

The cumulative incidence of renal damage was estimated using the Kaplan–Meier method and compared using the log-rank test. Three Cox proportional hazards regression models were constructed to determine the independent predictive value of CVAI for renal damage. Model 1 was adjusted for age and sex. Model 2 was adjusted for variables with significant differences among CVAI quartile groups, including age, sex, smoking status, drinking status, SBP, baseline eGFR, duration of hypertension, types of AGM, antidiabetic drugs, antihypertensive drugs, HbA1c, BUN, and hyperuricemia. Model 3 was adjusted for all included factors, including PAC, which has been recently shown to be independently associated with incident renal damage in hypertensives with AGM (20). Hazard ratios (HRs) for outcomes were calculated for quartiles CVAI (with the first quartile as reference), high CVAI (with the group below the median of CVAI as reference), and each SD increase of CVAI. The tolerance and VIF were used for collinearity testing among the included variables.

To evaluate the robustness of the results, sensitivity analyses were performed by excluding patients with a follow-up time of less than 12 months. Furthermore, interaction terms were introduced into the multivariable model to evaluate whether the association between CVAI and renal damage differed according to age (<60 or ≥60 years), sex (men or women), types of AGM (prediabetes or diabetes), SBP (<140 or ≥140 mmHg), DBP (<90 or ≥90 mmHg), BMI (<28 or ≥28 kg/m^2^), and medication use (antihypertensive, lipid-lowering, and hypoglycemic drugs).

The lack of repeated renal function measurements may have overestimated the outcomes of renal damage. Therefore, we used more stringent criteria to redefine the outcome as “overt renal damage” (eGFR < 50 ml/min/1.73 m^2^ and/or urine protein ≥ 2+), and repeated analyses were performed using the abovementioned procedure.

To describe the shape of the association between CVAI and incident renal damage, we used restricted cubic splines incorporated into the Cox models. In addition, the predictive power of the CVAI was examined and directly compared with other indices, including the VAI, BMI, WC, and WHtR, using the area under the receiver operating characteristic curve (AUC) and C-index. Statistical analyses were performed using Statistical Package for the Social Sciences (SPSS) version 23.0 for Windows (SPSS Inc., Chicago, IL, USA) and R version 4.0.3.

## Results

### Characteristics of the Study Population

In total, 2,033 participants with hypertension and AGM were finally included in the analysis. The mean age of the study population was 55 ± 11 years, and 884 (43.5%) patients were women. The mean SBP and DBP levels were 149 ± 21 and 88 ± 15 mmHg, respectively. The baseline eGFR was 118 ± 30 ml/min/1.73 m^2^. The median CVAI score was 154 (IQR: 129-182). Details of the baseline characteristics across the CVAI quartiles are shown in **Table 1**. Participants with higher CVAI levels tended to have higher BMI, WC, BP, HbA1c, TG, and BUN levels. In addition, with an increase in CVAI, there was an increased proportion of men, smokers, drinkers, hyperuricemia, and use of antidiabetic and antihypertensive drugs.

**Table 1:** Baseline characteristics of study population across CVAI quartiles.

Characteristics	Q1 (*n* = 508) 38.63–128.92	Q2 (*n* = 508) 128.93–154.03	Q3 (*n* = 508) 154.11–181.42	Q4 (*n* = 509) 181.56–376.25	*p*-Value
Age (year)	53.4 ± 10.2	56.8 ± 11.0	56.4 ± 11.5	55.5 ± 11.1	<0.001
Women, *n* (%)	344 (67.7)	272 (53.5)	182 (35.8)	86 (16.9)	<0.001
Ethnicity, *n* (%)					
Han	370 (72.8)	325 (64.0)	310 (61.0)	210 (41.3)	<0.001
Others	138 (27.2)	183 (36.0)	198 (39.0)	299 (58.7)	
BMI (kg/m^2^)	24.9 ± 2.5	27.0 ± 2.7	28.5 ± 3.0	31.8 ± 3.7	<0.001
WC (cm)	90.0 ± 7.2	97.4 ± 5.6	103.4 ± 6.0	113.6 ± 8.4	<0.001
Duration of HTN (year)	5.0 (2.0–10.0)	8.0 (3.0–13.0)	7.0 (2.0–13.0)	8.0 (3.0–13.0)	<0.001
SBP (mmHg)	146.9 ± 22.0	146.9 ± 21.1	147.8 ± 20.1	152.5 ± 21.1	<0.001
DBP (mmHg)	86.7 ± 14.3	85.6 ± 14.3	87.6 ± 14.3	91.7 ± 15.5	<0.001
AGM types, *n* (%)					
Prediabetes	260 (51.2)	216 (42.5)	183 (36.0)	193 (37.9)	<0.001
Diabetes	248 (48.8)	292 (57.5)	325 (64.0)	316 (62.1)	
HbA1c	6.7 ± 1.2	6.9 ± 1.3	7.0 ± 1.3	7.1 ± 1.4	<0.001
Smoking, *n* (%)	95 (18.7)	115 (22.6)	160 (31.5)	222 (43.6)	<0.001
Alcohol drinking, *n* (%)	82 (16.1)	115 (22.6)	155 (30.5)	187 (36.7)	<0.001
Total cholesterol (mmol/L)	4.44 ± 1.13	4.45 ± 1.05	4.37 ± 1.09	4.48 ± 1.12	0.489
Triglyceride (mmol/L)	1.40 (1.06–1.92)	1.70 (1.26–2.45)	1.72 (1.22–2.50)	1.91 (1.45–2.70)	<0.001
HDL-C (mmol/L)	1.07 ± 0.28	0.99 ± 0.23	0.94 ± 0.20	0.89 ± 0.19	<0.001
LDL-C (mmol/L)	2.67 ± 0.86	2.59 ± 0.85	2.60 ± 0.84	2.64 ± 0.88	0.449
Serum creatinine (μmol/L)	61.3 ± 15.1	63.9 ± 14.4	67.8 ± 16.1	71.2 ± 15.3	<0.001
Baseline eGFR (ml/min/1.73 m^2^)	119.6 (103.0–139.3)	115.7 (98.9–136.2)	112.9 (94.5–134.9)	110.7 (93.2–130.4)	<0.001
Blood urea nitrogen (mmol/L)	4.88 ± 1.35	5.05 ± 1.40	5.25 ± 1.52	5.24 ± 1.29	<0.001
Uric acid (μmol/L)	299.9 ± 77.3	331.0 ± 80.3	343.6 ± 85.2	354.4 ± 88.3	<0.001
Hyperuricemia, *n* (%)	69 (13.6)	116 (22.8)	120 (23.6)	131 (25.7)	<0.001
PAC (ng/dl)	13.5 (11.5–19.7)	13.9 (11.6–20.5)	13.6 (11.7–18.9)	13.6 (11.7–19.9)	0.627
PRA (ng/ml/h)	1.31 (0.51–2.47)	1.52 (0.57–2.67)	1.22 (0.45–2.64)	1.40 (0.60–2.69)	0.088
Antidiabetic drugs	233 (45.9)	284 (55.9)	306 (60.2)	315 (61.9)	<0.001
Lipid-lowering drugs	399 (78.5)	427 (84.1)	417 (82.1)	424 (83.3)	0.104
Anti-hypertensive drugs					
ACEI/ARB	261 (51.4)	292 (57.5)	293 (57.7)	329 (64.6)	<0.001
CCB	398 (78.3)	418 (82.3)	417 (82.1)	444 (87.2)	0.003
Beta-blocker	79 (15.6)	112 (22.0)	104 (20.5)	148 (29.1)	<0.001
Diuretics	159 (31.3)	170 (33.5)	190 (37.4)	197 (38.7)	0.049
Follow-up time (person-years)	1587	1431	1496	1436	−
Outcome incidence, number (incidence per 1000 person-years of follow-up)					
Renal damage	50 (31.5)	70 (48.9)	85 (56.8)	97 (67.5)	<0.001
Overt renal damage	14 (8.8)	25 (17.5)	32 (21.4)	46 (32.0)	<0.001

Data are presented as the mean ± SD, n (%), or median (interquartile range).

CVAI, Chinese visceral adiposity index; BMI, body mass index; WC, waist circumference; SBP, systolic blood pressure; DBP, diastolic blood pressure; AGM, abnormal glucose metabolism; HbA1c, glycosylated hemoglobin; HDL-C, high-density lipoprotein cholesterol; LDL-C, low-density lipoprotein cholesterol; eGFR, estimated glomerular filtration rate; PAC, plasma aldosterone concentration; PRA, plasma renin activity; ACEI, angiotensin-converting enzyme inhibitors; ARB, angiotensin receptor blockers; CCB, calcium channel blockers.

**Figure 1:**
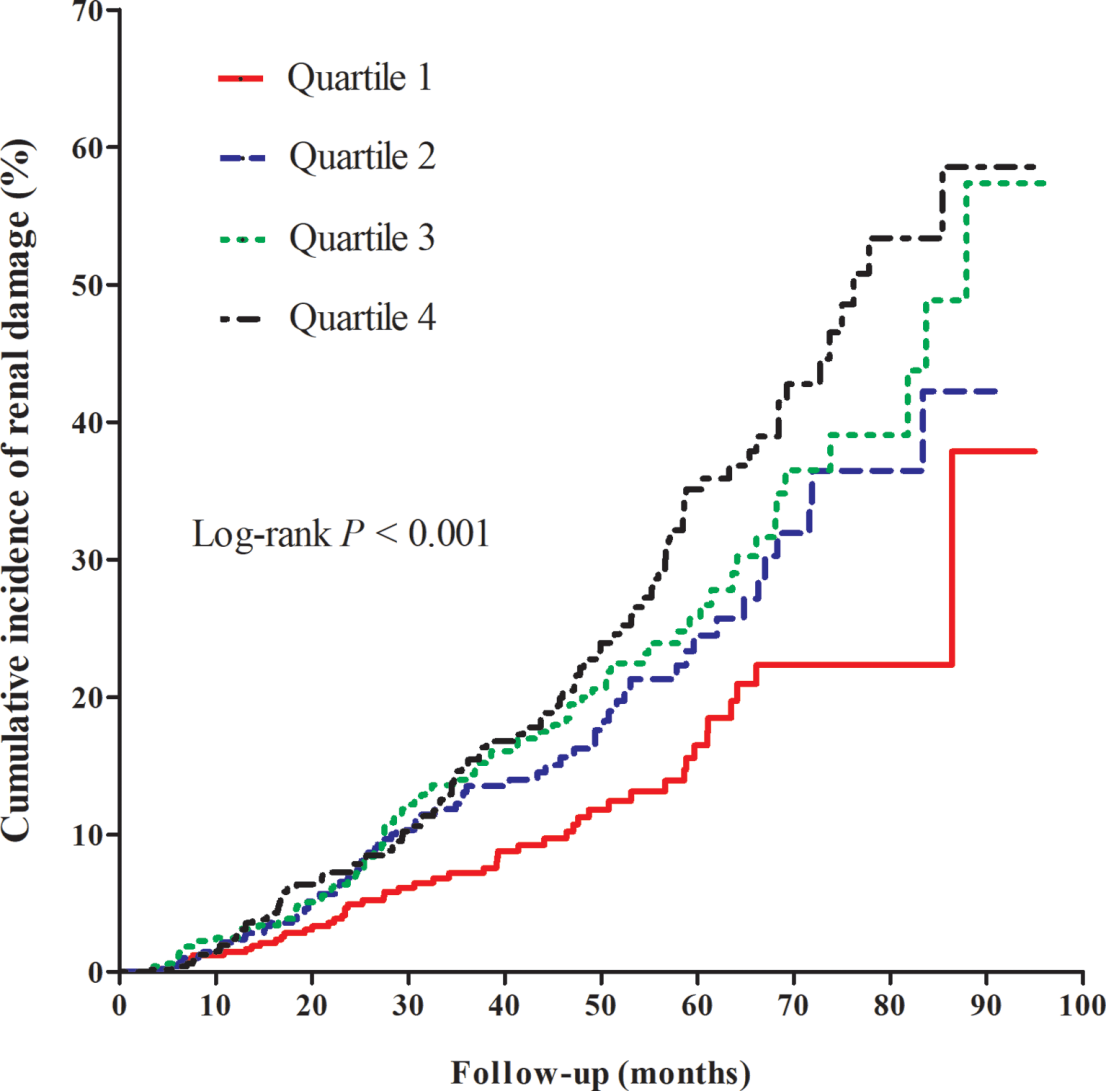
Kaplan–Meier curve of cumulative incidence of renal damage across quartiles of Chinese visceral adiposity index.

With a median follow-up of 2.6 (IQR: 1.5–4.2) years, the incidence of renal damage was 31.5, 48.9, 56.8, and 67.5/1,000 person-years across quartiles of CVAI. Regarding the outcome of overt renal damage (eGFR < 50 and/or urine protein ≥ 2+), a similar trend was observed (**Table 1**). The cumulative incidence of renal damage significantly increased with increasing CVAI (**Figure 1**), similar to the outcome of overt renal damage.

### Baseline Chinese Visceral Adiposity Index and Risk of Renal Damage

**Table 2** shows that the risk of renal damage significantly increased with increasing CVAI quartiles. After adjustment for potential confounders in Model 2, there was a significantly increased risk of incident renal damage for quartile 3 and quartile 4 of CVAI, with HRs and 95% CIs of 1.60 (1.11–2.31) and 1.70 (1.15–2.51), respectively. When all variables were adjusted (model 3), including PAC and PRA, consistent results were observed. Reanalyses by redefining the outcome as overt renal damage showed a stronger association (**Table 3**). The HRs (95% CIs) for overt renal damage in quartiles 2, 3, and 4 of CVAI were 1.85 (0.93–3.67), 2.36 (1.21–4.60), and 2.94 (1.47–5.89), respectively. Each SD increase in CVAI (SD = 42) had a 38% increased risk of overt renal damage. Consistent trends were observed in diabetes and prediabetes groups (**Tables S1, S2**), as well as separated by sex (**Table S3**). No obvious collinearity was detected among the variables in the fully adjusted models (**Table S4**).

**Table 2:** Multivariable Cox regression for the association between CVAI and incident renal damage.

CVAI	Crude model	*p*-Value	Model 1	*p*-Value	Model 2	*p*-Value	Model 3	*p*-Value
	HR (95% CI)		HR (95% CI)		HR (95% CI)		HR (95% CI)	
Quartile groups								
Quartile 1	Ref.		Ref.		Ref.		Ref.	
Quartile 2	1.61 (1.12–2.31)	0.010	1.55 (1.08–2.24)	0.018	1.42 (0.97–2.06)	0.069	1.36 (0.93–1.97)	0.111
Quartile 3	1.83 (1.29–2.60)	0.001	1.79 (1.25–2.56)	0.002	1.60 (1.11–2.31)	0.012	1.57 (1.09–2.27)	0.016
Quartile 4	2.16 (1.54–3.04)	<0.001	2.10 (1.46–3.03)	<0.001	1.70 (1.15–2.51)	0.008	1.65 (1.11–2.44)	0.013
*p* for trend		<0.001		<0.001		0.008		0.011
Dichotomous groups								
Lower (<154.1)	Ref.		Ref.		Ref.		Ref.	
Higher (≥ 154.1)	1.56 (1.23–1.96)	<0.001	1.51 (1.18–1.93)	0.001	1.34 (1.04–1.73)	0.024	1.34 (1.04–1.73)	0.025
Each SD increase	1.23 (1.11–1.38)	<0.001	1.22 (1.08–1.38)	0.002	1.13 (0.98–1.29)	0.085	1.12 (0.98–1.28)	0.112

Results are shown as hazard ratios (95% CIs) derived from Cox proportional hazards models. Model 1 was adjusted for age and sex. Model 2 was adjusted for age, sex, ethnicity, smoking status, drinking status, SBP, baseline eGFR, duration of hypertension, type of AGM, duration of AGM, antidiabetic drugs, antihypertensive drugs, HbA1c, BUN, and hyperuricemia. Model 3 was adjusted for variables in model 2 plus TC, LDL-C, lipid-lowering drugs, Ln PAC, and Ln PRA.

CVAI, Chinese visceral adiposity index; SBP, systolic blood pressure; eGFR, estimated glomerular filtration rate; AGM, abnormal glucose metabolism; HbA1c, glycosylated hemoglobin; BUN, blood urea nitrogen; TC, total cholesterol; LDL-C, low-density lipoprotein cholesterol; PAC, plasma aldosterone concentration; PRA, plasma renin activity.

**Table 3:** Multivariable Cox regression for the association between CVAI and incident overt renal damage.

CVAI	Crude model	*p*-Value	Model 1	*p*-Value	Model 2	*p*-Value	Model 3	*p*-Value
	HR (95% CI)		HR (95% CI)		HR (95% CI)		HR (95% CI)	
Quartile 1	Ref.		Ref.		Ref.		Ref.	
Quartile 2	2.05 (1.07–3.95)	0.032	2.05 (1.06–3.96)	0.033	1.98 (1.00–3.92)	0.050	1.85 (0.93–3.67)	0.079
Quartile 3	2.45 (1.31–4.60)	0.005	2.57 (1.35–4.90)	0.004	2.39 (1.23–4.64)	0.010	2.36 (1.21–4.60)	0.012
Quartile 4	3.64 (2.00–6.63)	<0.001	3.97 (2.10–7.51)	<0.001	3.01 (1.53–5.95)	0.002	2.94 (1.47–5.89)	0.002
*p* for trend		<0.001		<0.001		0.002		0.002
Dichotomous groups								
Lower (<154.1)	Ref.		Ref.		Ref.		Ref.	
Higher (≥ 154.1)	2.04 (1.39–3.00)	<0.001	2.10 (1.39–3.16)	<0.001	1.75 (1.15–2.68)	0.010	1.77 (1.15–2.72)	0.009
Each SD increase	1.46 (1.23–1.73)	<0.001	1.51 (1.25–1.83)	<0.001	1.39 (1.12–1.71)	0.002	1.38 (1.15–1.71)	0.003

Results are shown as hazard ratios (95% CIs) derived from Cox proportional hazards models. Overt renal damage was defined as an eGFR < 50 and/or urine protein ≥ 2+. Model 1 was adjusted for age and sex. Model 2 was adjusted for age, sex, ethnicity, smoking status, drinking status, SBP, baseline eGFR, duration of hypertension, type of AGM, duration of AGM, antidiabetic drugs, antihypertensive drugs, HbA1c, BUN, and hyperuricemia. Model 3 was adjusted for variables in model 2 plus TC, LDL-C, lipid-lowering drugs, Ln PAC, and Ln PRA.

CVAI, Chinese visceral adiposity index; eGFR, estimated glomerular filtration rate; SBP, systolic blood pressure; AGM, abnormal glucose metabolism; HbA1c, glycosylated hemoglobin; BUN, blood urea nitrogen; TC, total cholesterol; LDL-C, low-density lipoprotein cholesterol; PAC, plasma aldosterone concentration; PRA, plasma renin activity.

**Figure 2:**
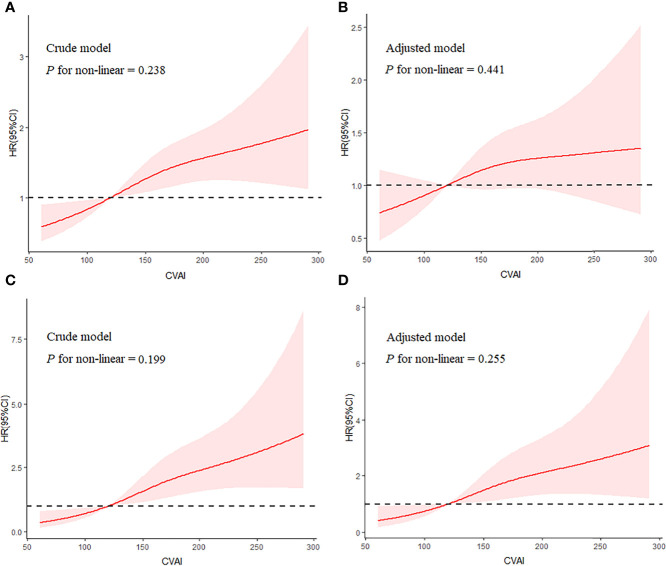
Shape of the association of CVAI with renal damage **(A, B)** and overt renal damage **(C, D)** by restricted cubic spline. Adjusted model included variables of age, sex, smoking status, drinking status, SBP, baseline eGFR, duration of hypertension, types of GMD, glucose metabolism disorders; antidiabetic drugs, anti-hypertension drugs, HbA1c, BUN, and hyperuricemia. CVAI, Chinese visceral adiposity index; SBP, systolic blood pressure; eGFR, estimated glomerular filtration rate; HbA1c, glycosylated hemoglobin; BUN, blood urea nitrogen.

By excluding participants with a follow-up time of less than 12 months, sensitivity analysis confirmed the robustness of the results (**Table S5**). In model 3, quartiles 3 and 4 of CVAI had 50% and 55% increased risks of incident renal damage, respectively (both *p* < 0.05). In addition, restricted cubic splines showed a linear dose–response association between CVAI and renal damage (*p*_nonlinearity_> 0.05, **Figure 2**).

### Subgroup Analyses and Prediction Power

**Figure 3:**
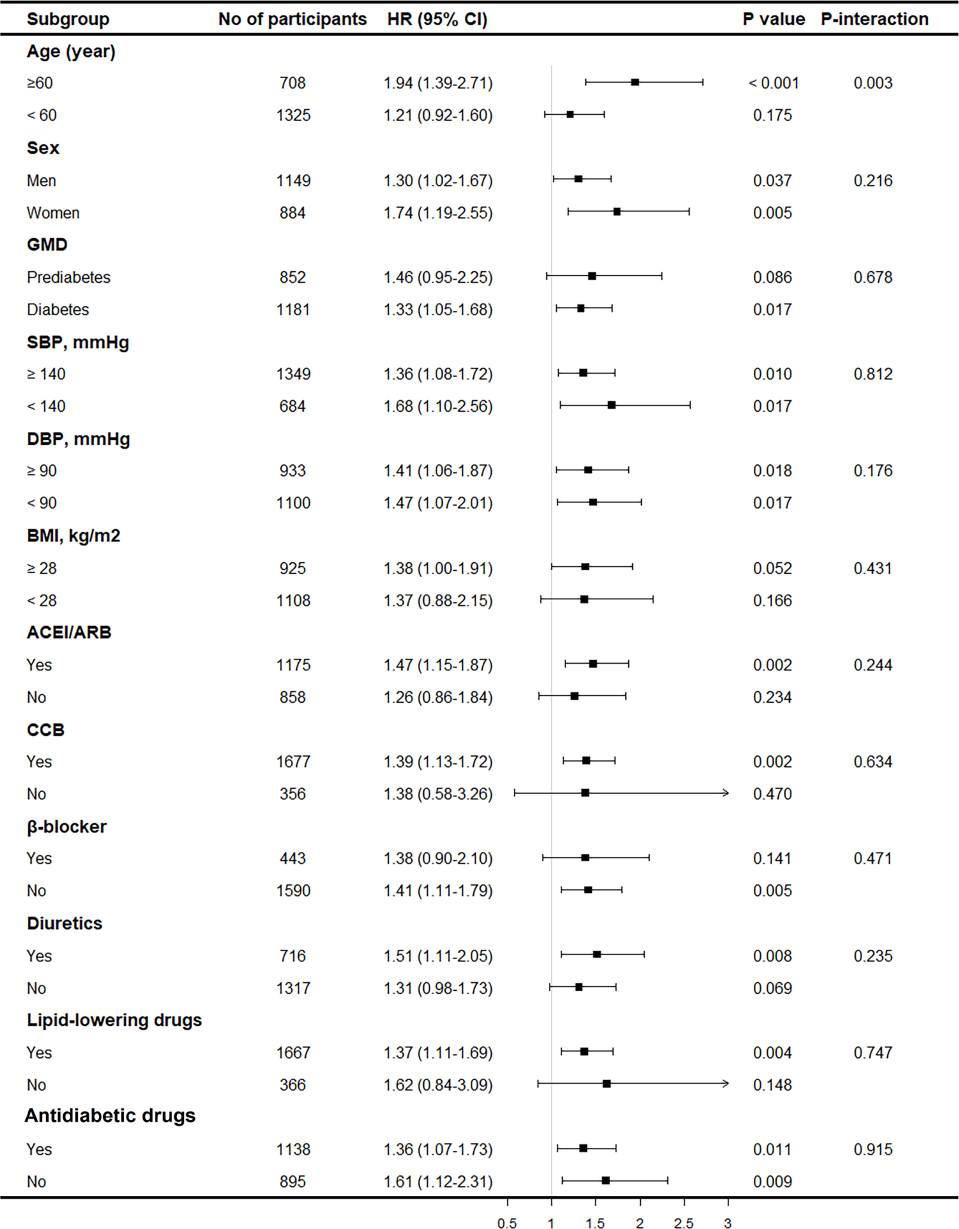
Subgroup analysis on the association between CVAI and overt renal damage. Results were derived from multivariable Cox regression adjusted for age, sex, smoking status, drinking status, SBP, baseline eGFR, duration of hypertension, types of GMD, glucose metabolism disorders; antidiabetic drugs, anti-hypertension drugs, HbA1c, BUN, and hyperuricemia and presented as hazard ratio for each SD increment of CVAI and the corresponding 95% CIs. CVAI, Chinese visceral adiposity index; SBP, systolic blood pressure; eGFR, estimated glomerular filtration rate; HbA1c, glycosylated hemoglobin; BUN, blood urea nitrogen.

Subgroup analyses were performed by age, sex, type of AGM, SBP, DBP, BMI, and medication use (antihypertensive, lipid-lowering, and antidiabetic drugs) to further evaluate the association between CVAI and renal damage. The results showed consistent trends in all subgroups for overt renal damage (**Figure 3**) and renal damage (**Figure S1**). In addition, none of the variables significantly modified the association (*p* for interaction >0.05), except for age (*p* for interaction < 0.05), and the association between CVAI and renal damage was stronger in older adults.

The AUC and C-index values of CVAI, VAI, BMI, WC, and WHtR for predicting incident renal damage are shown in **Table S6**. Among these obesity indices, the CVAI had the highest AUC and C-index, significantly higher than BMI, WC, and WHtR. By using ROC analysis, the best cutoff value for CVAI to distinguish individuals with and without incident renal damage was 149. In addition, when the VAI was compared with other indicators (BMI, WC, and WHtR), there was no significant difference (*p* > 0.05).

## Discussion

In the present study, with a longitudinal design, CVAI was positively associated with incident renal damage in a linear dose–response pattern in patients with hypertension and AGM. Furthermore, CVAI had the best performance in predicting incident renal damage compared with other obesity indices, including VAI, BMI, WC, and WHtR. Patients with hypertension and AGM tend to have a higher risk of kidney disease; however, most patients are already in an irreversible stage at the time of detection for CKD and usually have complications, and some of them even need renal replacement therapy (21). According to our study, for those with hypertension and AGM, a simple assessment of visceral adiposity by calculating CVAI may be helpful for the early identification of high-risk individuals. It is necessary to pay close attention to the high risk of renal damage when CVAI is greater than 149 and to adopt strict BP and glucose management, thereby reducing the risk of renal damage.

Obesity, especially visceral obesity, is associated with the occurrence and development of kidney disease (22, 23). Non-alcoholic fatty liver disease development and fibrosis progression have recently been shown to be associated with incident CKD (24). Although MRI and CT are the gold standard for measuring visceral fat, these techniques are rarely available in daily practice because of the limitations of equipment and cost. Several simple indicators such as BMI, WC, WHtR, and VAI are commonly used to assess obesity and fat distribution (25). However, body fat distribution varies by race, and it has been reported that the Asian population seems to be more inclined to visceral fat accumulation at a lower BMI (26). Previous studies have shown that VAI is not superior to BMI or WC in estimating visceral adipose tissue and predicting type 2 diabetes in the Chinese population (27). Similar results were observed in our study, with no significant difference in the predictive power of VAI for renal damage compared with BMI, WC, and WHtR. CVAI was initially established as a reliable indicator for evaluating metabolic health in the Chinese population and was further confirmed to be a strong and independent predictor of diabetes in Chinese adults (28, 29). In a recent cross-sectional study, CVAI showed the strongest association with cardiovascular disease among the commonly used abdominal obesity indices (30). Similarly, several other studies have demonstrated that CVAI is related to cardiovascular risk or its risk factors, such as carotid atherosclerosis (31–33). Our study extends this field by demonstrating an association between CVAI and incident renal damage.

The association between obesity and kidney disease has been reported in the general population and the population without diabetes (10, 14, 15, 34); however, as a reliable measure of visceral fat, the association between CVAI and kidney disease remains to be verified. To our knowledge, this is the first longitudinal study to evaluate the association between CVAI and the risk of renal damage in patients with hypertension and AGM. Based on previous studies and our analysis, several underlying mechanisms may be involved. First, it is mediated by BP and glucose. Given the association between visceral fat and other risk factors for kidney disease, higher CVAI may increase the risk of renal damage by exacerbating these factors, such as BP and glucose, especially in patients with hypertension and AGM. Second, a chronic inflammatory reaction may be involved. A higher CVAI represents an increased accumulation of visceral fat, which produces a variety of pro-inflammatory factors, such as tumor necrosis factor α, interleukin-6, and interleukin-8, resulting in the occurrence of renal damage (35, 36). Third, fat had a direct effect. The infiltration and accumulation of adipokines, produced by visceral adipose tissue, may induce structural and functional changes in podocytes and proximal tubule cells that contribute to renal damage (37, 38). Fourth, there are synergistic effects of multiple factors. High TG and WC and low HDL-C levels have been associated with kidney disease (39–41). Therefore, the stronger association between CVAI and renal damage may be partly explained by the synergistic effects of these factors.

By comparing the CVAI with other commonly used obesity indicators (BMI, WC, WHtR, and VAI), we found that the CVAI had the highest predictive power for renal damage. It has been reported that BMI cannot adequately discriminate between body fat mass and lean tissues or identify regional body fat distribution (42, 43). WC and WHtR can better reflect abdominal obesity than BMI but have limitations in distinguishing subcutaneous from visceral adipose tissue (44, 45). Interestingly, in our study, although no significant differences in AUC and C-index were observed between CVAI and VAI, the performance of VAI was not significantly improved when compared with the other three indices (BMI, WC, and WHtR, *p* > 0.05). This may also reconfirm that CVAI is more suitable than VAI for the Chinese population.

The present study has several strengths. First, a longitudinal design with a large sample size and a series of confounder adjustments yielded relatively stable and reliable results. Second, our study consisted of a sample of individuals at a high risk of renal damage, and the results may contribute to the prevention and treatment of kidney disease. However, several limitations of this study warrant discussion. First, single measurements of serum creatinine and urine protein without repeated examinations may have resulted in the misclassification of individuals with renal damage. Also, proteinuria was examined through qualitative but not quantitative methods. However, analyses by redefining the outcome as overt renal damage (eGFR < 50 and/or urine protein ≥ 2+) confirmed the robustness of the results. Second, although a wide range of confounders were adjusted, residual confounding factors were not considered, such as dietary and inflammation indicators. Also, future studies with larger sample sizes are needed to assess the association between diabetes and prediabetes separately. Third, the study was conducted in a single center, although it was conducted in a regional center for patients with hypertension of a large age range and ethnic groups. Fourth, using a retrospective design, we were unable to evaluate the association between the dynamic changes in CVAI and renal damage.

In conclusion, higher CVAI is associated with an increased risk of renal damage in patients with hypertension and AGM. Furthermore, CVAI has the best performance in predicting incident renal damage as compared to other obesity indices. Therefore, a simple assessment of visceral adiposity by calculating CVAI may be helpful for the early identification of high-risk individuals and adopting strict BP and glucose management, thereby reducing the risk of renal damage.

## Data Availability Statement

The original contributions presented in the study are included in the article/**Supplementary Material**. Further inquiries can be directed to the corresponding author.

## Ethics Statement

The studies involving human participants were reviewed and approved by People’s Hospital of Xinjiang Uygur Autonomous Region. The patients/participants provided their written informed consent to participate in this study.

## Author Contributions

MYL contributed to the study design and statistical analysis. MYL, NL, MH, and LG analyzed the data together and drafted the manuscript. QZ, LY, ML, and WY participated in the data collection. All authors have read and approved the final manuscript.

## Funding

This work was supported by the Non-Profit Central Research Institute Fund of the Chinese Academy of Medical Sciences (grant number 2020-RW330-002).

## Conflict of Interest

The authors declare that the research was conducted in the absence of any commercial or financial relationships that could be construed as a potential conflict of interest.

## Publisher’s Note

All claims expressed in this article are solely those of the authors and do not necessarily represent those of their affiliated organizations, or those of the publisher, the editors and the reviewers. Any product that may be evaluated in this article, or claim that may be made by its manufacturer, is not guaranteed or endorsed by the publisher.
