# Supplementary material for: Chinese Visceral Adiposity Index Is Associated With Incident Renal Damage in Patients With Hypertension and Abnormal Glucose Metabolism: A Longitudinal Study
Source: Front Endocrinol (Lausanne). 2022 Jul 14;13:910329. doi: 10.3389/fendo.2022.910329 (PMC9329673; doi:10.3389/fendo.2022.910329)
Supplement: Supplementary file 1 [file DataSheet_1.docx]

**Supplementary materials**

**Chinese visceral adiposity index is associated with incident renal damage in patients with hypertension and abnormal glucose metabolism: a longitudinal study**

**Short title:** CVAI and renal damage

**All author’s names:** Mengyue Lin^1^, Nanfang Li^1^, Mulalibieke Heizhati^1^, Lin Gan^1^, Qing Zhu^1^, Ling Yao^1^, Mei Li^1^, Wenbo Yang^1^

**Author’s affiliations:**

^1^ Hypertension Center of People’s Hospital of Xinjiang Uygur Autonomous Region; Xinjiang Hypertension Institute; National Health Committee Key Laboratory of Hypertension Clinical Research; Key Laboratory of Xinjiang Uygur Autonomous Region “Hypertension Research Laboratory; Xinjiang Clinical Medical Research Center for Hypertension (Cardio-Cerebrovascular) Diseases.

**Correspondence to:** Professor Nanfang Li, Hypertension Center of People’s Hospital of Xinjiang Uygur Autonomous Region, 91 Tianchi Road, Urumqi, Xinjiang 830001, China. E-mail: [lnanfang2016@sina.com](mailto:lnanfang2016@sina.com).

**Table S1** Multivariable Cox regression for the association between CVAI and incident renal damage (separated by types of AGM)

| CVAI | Crude model | *P* value | Model 1 | *P* value | Model 2 | *P* value | Model 3 | *P* value |
| --- | --- | --- | --- | --- | --- | --- | --- | --- |
|  | HR (95% CI) |  | HR (95% CI) |  | HR (95% CI) |  | HR (95% CI) |  |
| **Diabetes** |  |  |  |  |  |  |  |  |
| Quartile 1 | Ref. |  | Ref. |  | Ref. |  | Ref. |  |
| Quartile 2 | 1.89 (1.21-2.96) | 0.005 | 1.83 (1.16-2.86) | 0.009 | 1.56 (0.98-2.47) | 0.062 | 1.50 (0.94-2.39) | 0.086 |
| Quartile 3 | 2.01 (1.31-2.82) | 0.001 | 1.94 (1.25-2.99) | 0.003 | 1.80 (1.15-2.82) | 0.010 | 1.81 (1.15-2.83) | 0.010 |
| Quartile 4 | 1.82 (1.18-2.82) | 0.007 | 1.68 (1.07-2.66) | 0.026 | 1.38 (0.85-2.25) | 0.195 | 1.39 (0.85-2.28) | 0.184 |
| *P* for trend |  | 0.014 |  | 0.044 |  | 0.229 |  | 0.191 |
| Dichotomous groups |  |  |  |  |  |  |  |  |
| Lower (< 154.1) | Ref. |  | Ref. |  | Ref. |  | Ref. |  |
| Higher (≥ 154.1) | 1.34 (1.02-1.77) | 0.038 | 1.29 (0.97-1.72) | 0.086 | 1.23 (0.91-1.66) | 0.185 | 1.26 (0.93-1.70) | 0.138 |
| Each SD increase | 1.15 (1.01-1.31) | 0.031 | 1.13 (0.98-1.30) | 0.103 | 1.07 (0.92-1.25) | 0.384 | 1.07 (0.92-1.25) | 0.363 |
| **Prediabetes** |  |  |  |  |  |  |  |  |
| Quartile 1 | Ref. |  | Ref. |  | Ref. |  | Ref. |  |
| Quartile 2 | 1.05 (0.55-2.01) | 0.874 | 1.10 (0.57-2.11) | 0.786 | 1.08 (0.55-2.12) | 0.827 | 1.04 (0.53-2.06) | 0.906 |
| Quartile 3 | 1.15 (0.60-2.20) | 0.665 | 1.36 (0.68-2.70) | 0.387 | 1.20 (0.59-2.45) | 0.619 | 1.20 (0.59-2.47) | 0.615 |
| Quartile 4 | 2.59 (1.50-4.47) | 0.001 | 3.17 (1.71-5.89) | < 0.001 | 2.62 (1.30-5.27) | 0.007 | 2.60 (1.26-5.36) | 0.010 |
| *P* for trend |  | 0.001 |  | < 0.001 |  | 0.007 |  | 0.010 |
| Dichotomous groups |  |  |  |  |  |  |  |  |
| Lower (< 154.1) | Ref. |  | Ref. |  | Ref. |  | Ref. |  |
| Higher (≥ 154.1) | 1.81 (1.19-2.75) | 0.006 | 2.06 (1.29-3.30) | 0.003 | 1.66 (1.00-2.76) | 0.052 | 1.67 (1.00-2.80) | 0.052 |
| Each SD increase | 1.29 (1.05-1.59) | 0.014 | 1.37 (1.09-1.72) | 0.008 | 1.22 (0.93-1.59) | 0.145 | 1.21 (0.92-1.60) | 0.164 |

Results are shown as hazard ratios (95% confidence intervals) derived from Cox proportional hazard models. Model 1 was adjusted for age and sex. Model 2 was adjusted for age, sex, ethnicity, smoking status, drinking status, SBP, baseline eGFR, duration of hypertension, type of AGM, duration of AGM, anti-diabetic drugs, anti-hypertensive drugs, HbA1c, BUN, and hyperuricemia. Model 3 was adjusted for variables in model 2 plus TC, LDL-C, lipid-lowering drugs, Ln PAC, and Ln PRA.

**Table S2** Multivariable Cox regression for the association between CVAI and incident overt renal damage (separated by types of AGM)

| CVAI | Crude model | *P* value | Model 1 | *P* value | Model 2 | *P* value | Model 3 | *P* value |
| --- | --- | --- | --- | --- | --- | --- | --- | --- |
|  | HR (95% CI) |  | HR (95% CI) |  | HR (95% CI) |  | HR (95% CI) |  |
| **Diabetes** |  |  |  |  |  |  |  |  |
| Quartile 1 | Ref. |  | Ref. |  | Ref. |  | Ref. |  |
| Quartile 2 | 2.15 (1.01-4.60) | 0.048 | 2.14 (1.00-4.58) | 0.051 | 1.97 (0.89-4.37) | 0.095 | 1.86 (0.84-4.12) | 0.128 |
| Quartile 3 | 2.49 (1.21-5.12) | 0.013 | 2.51 (1.21-5.20) | 0.013 | 2.57 (1.20-5.51) | 0.015 | 2.55 (1.19-5.49) | 0.016 |
| Quartile 4 | 2.68 (1.31-5.48) | 0.007 | 2.75 (1.30-5.82) | 0.008 | 2.30 (1.03-5.14) | 0.042 | 2.36 (1.05-5.31) | 0.039 |
| *P* for trend |  | 0.008 |  | 0.009 |  | 0.046 |  | 0.034 |
| Dichotomous groups |  |  |  |  |  |  |  |  |
| Lower (< 154.1) | Ref. |  | Ref. |  | Ref. |  | Ref. |  |
| Higher (≥ 154.1) | 1.66 (1.07-2.58) | 0.024 | 1.68 (1.06-2.65) | 0.027 | 1.61 (0.99-2.61) | 0.056 | 1.67 (1.02-2.72) | 0.040 |
| Each SD increase | 1.33 (1.10-1.62) | 0.004 | 1.37 (1.11-1.69) | 0.004 | 1.35 (1.06-1.70) | 0.013 | 1.37 (1.08-1.73) | 0.010 |
| **Prediabetes** |  |  |  |  |  |  |  |  |
| Quartile 1 | Ref. |  | Ref. |  | Ref. |  | Ref. |  |
| Quartile 2 | 1.56 (0.42-5.79) | 0.510 | 1.77 (0.47-6.68) | 0.402 | 1.53 (0.40-5.95) | 0.537 | 1.92 (0.47-7.90) | 0.367 |
| Quartile 3 | 1.35 (0.34-5.40) | 0.672 | 1.93 (0.45-8.29) | 0.379 | 1.34 (0.30-5.88) | 0.701 | 1.84 (0.39-8.78) | 0.443 |
| Quartile 4 | 5.72 (1.91-17.14) | 0.002 | 8.87 (2.58-30.47) | 0.001 | 4.42 (1.16-16.80) | 0.029 | 5.73 (1.31-24.99) | 0.020 |
| *P* for trend |  | 0.001 |  | < 0.001 |  | 0.025 |  | 0.019 |
| Dichotomous groups |  |  |  |  |  |  |  |  |
| Lower (< 154.1) | Ref. |  | Ref. |  | Ref. |  | Ref. |  |
| Higher (≥ 154.1) | 2.78 (1.27-6.11) | 0.011 | 3.54 (1.46-8.58) | 0.005 | 2.06 (0.79-5.36) | 0.140 | 2.36 (0.84-6.64) | 0.105 |
| Each SD increase | 1.62 (1.15-2.28) | 0.006 | 1.80 (1.23-2.62) | 0.002 | 1.41 (0.88-2.29) | 0.154 | 1.51 (0.90-2.53) | 0.121 |

Results are shown as hazard ratios (95% confidence intervals) derived from Cox proportional hazard models. Overt renal damage was defined as an eGFR <50 and/or urine protein ≥2+. Model 1 was adjusted for age and sex. Model 2 was adjusted for age, sex, ethnicity, smoking status, drinking status, SBP, baseline eGFR, duration of hypertension, type of AGM, duration of AGM, anti-diabetic drugs, anti-hypertensive drugs, HbA1c, BUN, and hyperuricemia. Model 3 was adjusted for variables in model 2 plus TC, LDL-C, lipid-lowering drugs, Ln PAC, and Ln PRA.

**Table S3** Multivariable Cox regression for the association between CVAI and incident overt renal damage (separated by sex)

| CVAI | Crude model | *P* value | Model 1 | *P* value | Model 2 | *P* value | Model 3 | *P* value |
| --- | --- | --- | --- | --- | --- | --- | --- | --- |
|  | HR (95% CI) |  | HR (95% CI) |  | HR (95% CI) |  | HR (95% CI) |  |
| **Men** |  |  |  |  |  |  |  |  |
| Quartile 1 | Ref. |  | Ref. |  | Ref. |  | Ref. |  |
| Quartile 2 | 2.03 (0.86-4.80) | 0.105 | 2.06 (0.87-4.85) | 0.100 | 2.30 (0.96-5.52) | 0.063 | 2.08 (0.86-5.03) | 0.104 |
| Quartile 3 | 2.46 (1.08-5.58) | 0.032 | 2.47 (1.09-5.61) | 0.031 | 2.45 (1.05-5.73) | 0.038 | 2.18 (0.92-5.16) | 0.077 |
| Quartile 4 | 4.12 (1.86-9.11) | < 0.001 | 3.99 (1.80-8.84) | 0.001 | 4.18 (1.80-9.75) | 0.001 | 3.89 (1.65-9.16) | 0.002 |
| *P* for trend |  | < 0.001 |  | < 0.001 |  | 0.001 |  | 0.002 |
| Dichotomous groups |  |  |  |  |  |  |  |  |
| Lower (< 154.1) | Ref. |  | Ref. |  | Ref. |  | Ref. |  |
| Higher (≥ 154.1) | 2.01 (1.13-3.55) | 0.017 | 1.99 (1.13-3.54) | 0.018 | 1.93 (1.06-3.53) | 0.031 | 1.88 (1.02-3.48) | 0.044 |
| Each SD increase | 1.40 (1.12-1.76) | 0.003 | 1.39 (1.11-1.75) | 0.005 | 1.39 (1.07-1.80) | 0.013 | 1.37 (1.05-1.79) | 0.019 |
| **Women** |  |  |  |  |  |  |  |  |
| Quartile 1 | Ref. |  | Ref. |  | Ref. |  | Ref. |  |
| Quartile 2 | 0.98 (0.32-3.04) | 0.969 | 1.06 (0.34-3.34) | 0.918 | 1.15 (0.33-4.01) | 0.829 | 1.01 (0.28-3.57) | 0.994 |
| Quartile 3 | 2.62 (1.01-6.76) | 0.047 | 2.89 (1.09-7.63) | 0.032 | 2.28 (0.77-6.75) | 0.137 | 2.13 (0.71-6.40) | 0.178 |
| Quartile 4 | 3.58 (1.44-8.87) | 0.006 | 4.18 (1.58-11.03) | 0.004 | 2.61 (0.86-7.89) | 0.089 | 2.40 (0.74-7.78) | 0.143 |
| *P* for trend |  | 0.001 |  | < 0.001 |  | 0.034 |  | 0.048 |
| Dichotomous groups |  |  |  |  |  |  |  |  |
| Lower (< 154.1) | Ref. |  | Ref. |  | Ref. |  | Ref. |  |
| Higher (≥ 154.1) | 2.35 (1.33-4.14) | 0.003 | 2.41 (1.34-4.33) | 0.003 | 1.57 (0.83-2.97) | 0.162 | 1.48 (0.76-2.87) | 0.246 |
| Each SD increase | 1.69 (1.28-2.23) | < 0.001 | 1.78 (1.33-2.38) | < 0.001 | 1.45 (1.05-1.99) | 0.024 | 1.42 (1.00-2.01) | 0.051 |

Results are shown as hazard ratios (95% confidence intervals) derived from Cox proportional hazard models. Overt renal damage was defined as an eGFR <50 and/or urine protein ≥2+. Model 1 was adjusted for age. Model 2 was adjusted for age, ethnicity, smoking status, drinking status, SBP, baseline eGFR, duration of hypertension, type of AGM, duration of AGM, anti-diabetic drugs, anti-hypertensive drugs, HbA1c, BUN, and hyperuricemia. Model 3 was adjusted for variables in model 2 plus TC, LDL-C, lipid-lowering drugs, Ln PAC, and Ln PRA.

**Table S4** Collinearity test of included variables

| Variables | Tolerance | Variance inflation factor |
| --- | --- | --- |
| CVAI | 0.701 | 1.427 |
| Age | 0.623 | 1.605 |
| Sex | 0.659 | 1.518 |
| Ethnicity | 0.844 | 1.184 |
| SBP | 0.867 | 1.153 |
| HbA1c | 0.686 | 1.457 |
| TC | 0.425 | 2.351 |
| LDL-C | 0.435 | 2.298 |
| Baseline eGFR | 0.728 | 1.373 |
| Duration of hypertension | 0.742 | 1.347 |
| ACEI/ARB | 0.842 | 1.187 |
| CCB | 0.907 | 1.103 |
| Beta blocker | 0.918 | 1.090 |
| Diuretics | 0.869 | 1.151 |
| Types of AGM | 0.528 | 1.895 |
| Duration of AGM | 0.697 | 1.435 |
| Anti-diabetic drugs | 0.520 | 1.925 |
| Lipid-lowering drugs | 0.938 | 1.066 |
| BUN | 0.791 | 1.265 |
| Hyperuricemia | 0.888 | 1.126 |
| Ln-PAC | 0.883 | 1.133 |
| Ln-PRA | 0.820 | 1.219 |

**Table S5** Multivariable Cox regression for the association between CVAI and incident renal damage^#^

| CVAI | Crude model | *P* value | Model 1 | *P* value | Model 2 | *P* value | Model 3 | *P* value |
| --- | --- | --- | --- | --- | --- | --- | --- | --- |
|  | HR (95% CI) |  | HR (95% CI) |  | HR (95% CI) |  | HR (95% CI) |  |
| Quartile 1 | Ref. |  | Ref. |  | Ref. |  | Ref. |  |
| Quartile 2 | 1.56 (1.05-2.31) | 0.028 | 1.49 (1.00-2.22) | 0.049 | 1.36 (0.89-2.01) | 0.166 | 1.28 (0.85-1.92) | 0.243 |
| Quartile 3 | 1.86 (1.27-2.72) | 0.001 | 1.79 (1.21-2.64) | 0.003 | 1.55 (1.04-2.31) | 0.031 | 1.50 (1.01-2.24) | 0.047 |
| Quartile 4 | 2.23 (1.54-3.22) | < 0.001 | 2.11 (1.43-3.13) | < 0.001 | 1.60 (1.05-2.44) | 0.029 | 1.55 (1.01-2.37) | 0.044 |
| *P* for trend |  | < 0.001 |  | < 0.001 |  | 0.026 |  | 0.036 |
| Dichotomous groups |  |  |  |  |  |  |  |  |
| Lower (< 154.1) | Ref. |  | Ref. |  | Ref. |  | Ref. |  |
| Higher (≥ 154.1) | 1.59 (1.24-2.03) | < 0.001 | 1.52 (1.17-1.98) | 0.002 | 1.32 (1.00-1.73) | 0.049 | 1.31 (1.00-1.72) | 0.053 |
| Each SD increase | 1.26 (1.12-1.41) | < 0.001 | 1.23 (1.08-1.40) | 0.002 | 1.12 (0.97-1.29) | 0.133 | 1.11 (0.96-1.28) | 0.167 |

^#^ Sensitivity analysis by excluding subjects with follow-up time less than 12 months.

Results are shown as hazard ratios (95% confidence intervals) derived from Cox proportional hazard models. Model 1 was adjusted for age and sex. Model 2 was adjusted for age, sex, ethnicity, smoking status, drinking status, SBP, baseline eGFR, duration of hypertension, type of AGM, duration of AGM, anti-diabetic drugs, anti-hypertensive drugs, HbA1c, BUN, and hyperuricemia. Model 3 was adjusted for variables in model 2 plus TC, LDL-C, lipid-lowering drugs, Ln PAC, and Ln PRA.

**Table S6** AUC and C-index for obesity indices

| Variables | AUC (95% CI) | *P* value |  | C-index (95% CI) | *P* value |
| --- | --- | --- | --- | --- | --- |
| CVAI | 0.574 (0.539-0.608) | Ref. |  | 0.561 (0.525-0.597) | Ref. |
| VAI | 0.563 (0.527-0.598) | 0.610 |  | 0.544 (0.506-0.583) | 0.241 |
| BMI | 0.548 (0.512-0.583) | 0.071 |  | 0.522 (0.484-0.560) | 0.005 |
| WC | 0.544 (0.509-0.580) | 0.001 |  | 0.533 (0.494-0.571) | 0.002 |
| WHtR | 0.541 (0.506-0.577) | 0.017 |  | 0.530 (0.491-0.568) | 0.017 |

AUC, area under receiver operating characteristic curves; CVAI, Chinese visceral adiposity index; VAI, visceral adiposity index; BMI, body mass index; WC, waist circumference; WHtR, waist-to-height ratio.


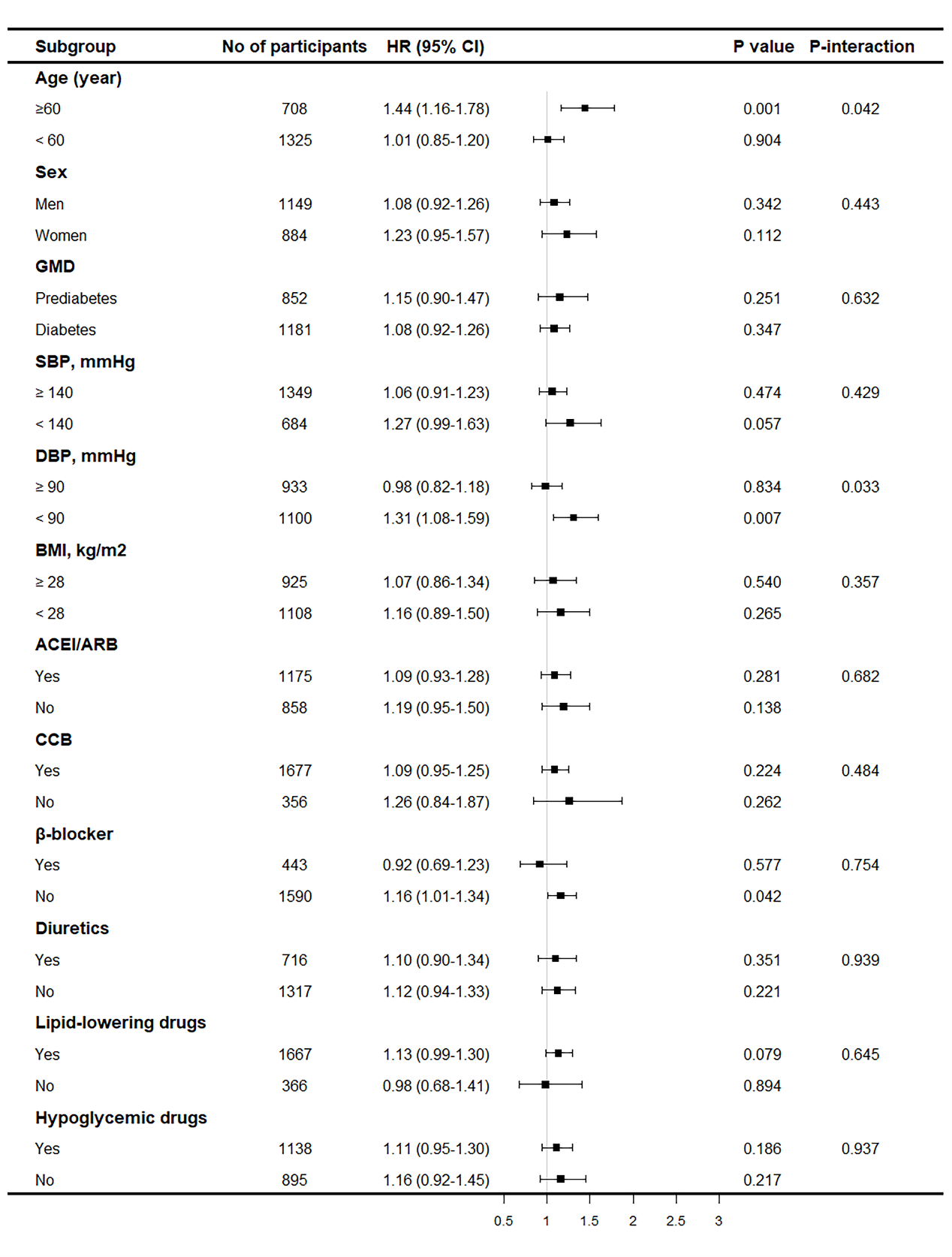


**Figure S1** Subgroup analysis on the association between CVAI and renal damage. Results were derived from multivariate Cox regression adjusted for age, sex, smoking status, drinking status, SBP, baseline eGFR, duration of hypertension, types of GMD, anti-diabetic drugs, anti-hypertension drugs, HbA1c, BUN, hyperuricemia, and presented as hazard ratio for each SD increment of CVAI and the corresponding 95% confidence intervals.
